# Editorial: Ethnography in the open science and digital age: new debates, dilemmas, and issues

**DOI:** 10.3389/fsoc.2024.1392012

**Published:** 2024-03-15

**Authors:** Colin Jerolmack, Alexandra K. Murphy, Victoria Reyes

**Affiliations:** ^1^Department of Environmental Studies and Sociology, New York University, New York, NY, United States; ^2^Department of Sociology, University of Michigan, Ann Arbor, MI, United States; ^3^Department of Gender and Sexuality Studies, University of California, Riverside, Riverside, CA, United States

**Keywords:** ethnography, data transparency, open science, digital ethnography, research methods, reflexivity

Ethnographers—and qualitative researchers more broadly—arguably face unprecedented challenges in carrying out their work today. Academic gatekeepers are increasingly demanding that fieldnotes, interview transcripts, and other data be shared in the name of “open science.” Popular ethnographies have had their scholarly rigor impugned by journalists and the public. Scrutiny has also come from within: As practitioners of the craft of ethnography come from more diverse backgrounds, some question longstanding conventions of writing, representation, and ethics. What's more, the digital and surveillance age poses novel challenges to how ethnographers study social life and protect the privacy of their participants.

While the “replication crisis” in social science has catalyzed a movement for transparency (e.g., registering hypotheses in advance; sharing data), it is not clear that positivistic standards of verification translate to the interpretive enterprise of ethnography (Jerolmack, 2023). Some, like Lubet (2018), contend that ethnographers must name sources, fact-check, and perhaps even share raw data to secure readers' trust and facilitate falsifiability; it has also been suggested that ethnographers who spurn open science may be left behind as funders and academic journals increasingly require data transparency (Jerolmack and Murphy, 2019). Others, like Burawoy (2017, p. 269), worry that the fetishization of “factual details” conveys a ”false sense of objectivity” that elides a reflexive reckoning with how our interpretations are shaped by our social position in the field; moreover, some (e.g., Reyes, 2018; Stuart, 2020) warn that demands for open science may further marginalize scholars who study vulnerable populations (where data transparency is dangerous) or who lack the resources that facilitate data transparency (e.g., hiring fact checkers). Between these poles, others have floated flexible “standards for transparency that are consistent” with ethnographers' “commitment to their subjects and interpretive scholarship” (Murphy et al., 2021, p. 41)—e.g., partial disclosure of people or places, sharing the coding scheme, or online appendices with supplementary data (Lee, 2016; Tsai et al., 2016; Reyes, 2018; Contreras, 2019)—and suggested criteria for evaluating scholarly rigor attuned to the *verstehen* spirit of qualitative methods (Small and Calarco, 2022).

As social life is increasingly lived online, it becomes unclear where the boundaries of the “field site” should be drawn and whether ethnographic conventions—methodological *and* ethical—are directly transferrable to the study of digital spaces (Lane, 2018; Stuart, 2020). Yet many contemporary ethnographies still read almost as if they were set in the prior millennium, barely acknowledging, much less theorizing, how much people have folded smart phones, social media, online gaming, virtual reality, and AI into their lives. As more researchers venture into digital spaces, they force us to grapple with questions like whether an online platform is a “community”—or even a “place”—and whether exchanging DMs or commenting on someone's post “counts” as ethnography.

With a growing chorus of social critics calling ethnography “extractive” and demanding that it be “decolonized,” also at issue is whom has license to write about whom, and what we owe our research participants (Rios, 2015; Miller, 2021). Relatedly, feminist ethnographers are calling for open, critical discussions about the embodied dimensions of fieldwork (a historically androcentric enterprise), including not only emotions but also issues like sexual intimacy and harassment (Hoang, 2015; Hanson and Richards, 2019; Reyes, 2020).

There can be no “one size fits all” answer to these developments and debates. This Research Topic therefore embraces a pluralistic view, curating a collection of methodological reflections that represent varying—even conflicting—perspectives on how ethnographers are engaging (or should engage) with the three pressing issues intimated above: the movement for open science; the migration of social life into digital spaces; and the moment of reckoning with the racialized and gendered history of fieldwork and knowledge production.

On the question of how ethnographers should respond to open science, two articles reject blanket demands for data transparency and question its value. Khan et al. contend that the college students whose sexual practices they studied would be less likely to disclose personal details, and that so much information would have to be masked to maintain confidentiality that the remaining data would be meaningless. Pugh and Mosseri contend that reflexivity is a better path to scholarly credibility and reliability than data transparency, and that unmasking participants' identities would pressure them into inauthentic performances of “narrative and emotional coherence.” (However, we note that one exemplar of “excavating ambivalence, plurality and complexity” *does* name—see Duneier, 1999). Taking a more meta critical approach, Goldensher makes the case for ethnographically studying open science as a contested field where gatekeepers (journal editors, grantors) privilege and legitimize certain forms of knowledge. Enriquez, a practitioner of open science, uses her experience making interviews with gig workers publicly available online to illustrate the kinds of ethical and practical issues involved with data sharing. As a journalist slightly removed from the open science debates, Conover puzzles over some of ethnography's conventions around confidentiality and data verification while appreciating that ethnographers have different commitments and face different pressures than journalists.

Regarding the study of digital spaces, two articles provide practical takeaways from observing the online world of adherents to the far-right conspiracy theory QAnon. By comparing Forberg's “digital ethnography” of QAnon to Schilt's “analog ethnography” of a different group, the authors conclude that the two modalities “share a common epistemology” and that the former can be as “thick” as the latter if the researcher commits to reflexive immersion (rather than just lurking). Regarding ethics, Cera argues that not all social media data should be treated as public and explores how to protect privacy while still making raw data accessible. The article by Owens unpacks the problem, exacerbated in online research, of how to deal with subjects who deceive us about their identity, experiences, or relationship to the field of research.

Becker (1967) long ago urged ethnographers to discard the myth of value neutrality. This imperative has taken on heightened urgency given the resurgence of nativism and racism. Ince rejects a “spectatorship” orientation to fieldwork in favor of the ethnographer as what James Baldwin called the *witness*, which requires “using one's status position to publicly unveil” structural inequality and advocate for change. In turn, Su and Su offer an *inward* perspective on reflexivity and the project of challenging social marginalization. The sisters reflect on how they responded to a shared experience of being sexually harassed in the field and suggest that we consider how such traumatic episodes shape the way we interpret the field—and ourselves.

The articles herein grapple with some of the most important dilemmas facing ethnographers today. These issues demand our scholarly attention, and the range of perspectives brought to bear upon them by the Research Topic's authors promise to bolster the craft of qualitative inquiry.

## Author contributions

CJ: Writing – review & editing, Writing – original draft. AM: Writing – review & editing. VR: Writing – review & editing.
